# Divergent Obesity Classification Between the Edmonton Obesity Staging System and the Lancet Commission Model

**DOI:** 10.1002/oby.70295

**Published:** 2026-09-01

**Authors:** Tobias Hagemann, Arya M. Sharma, Matthias Blüher, Anne Hoffmann

**Affiliations:** ^1^ Helmholtz Institute for Metabolic, Obesity and Vascular Research (HI‐MAG) of the Helmholtz Zentrum München at the University of Leipzig and University Hospital Leipzig Leipzig Germany; ^2^ Department of Medicine University of Alberta Edmonton Alberta Canada; ^3^ Divisions of Endocrinology and Nephrology, Leipzig University Medical Center University of Leipzig Leipzig Germany; ^4^ LeiCeM ‐ Leipzig Center of Metabolism Leipzig University Leipzig Germany

**Keywords:** BMI, clinical obesity, Edmonton Obesity Staging System, Lancet Commission, preclinical obesity

## Abstract

**Objective:**

BMI alone does not capture obesity‐related health heterogeneity. The Edmonton Obesity Staging System (EOSS) grades obesity severity based on comorbidities and functional impairment, whereas the Lancet Commission Diagnostic Model for Obesity (DMO) distinguishes preclinical from clinical obesity based on organ dysfunction. We compared classification patterns and concordance across both obesity frameworks in a large population‐based cohort.

**Methods:**

A modified EOSS and DMO were applied to the UK Biobank (*N* ≈ 411,000). Stage distributions, cross‐classification, and the impact of combining BMI with fat distribution on obesity categorization were analyzed.

**Results:**

About one‐quarter of participants were classified with obesity under both frameworks. Most were assigned to advanced stages, with high concordance for established disease. Differences were most pronounced in early stages: DMO captured a broader spectrum of mild/subclinical organ dysfunction, whereas EOSS emphasized more advanced manifestations of obesity‐related disease. Discrepancies reflected differences in operationalization of, for example, metabolic, cardiovascular, and mental health domains. Obesity thresholds influenced classification, with ~50% reclassified when BMI was combined with different fat distribution parameters, highlighting the sensitivity of early‐stage assignment.

**Conclusions:**

EOSS and DMO provide complementary approaches to obesity classification, with differing classification patterns highlighting distinct concepts of obesity‐related disease severity.

## Study Importance

- What is already known?
  ○ BMI alone poorly reflects obesity‐related health risk; comorbidities, organ dysfunction, and functional impairments are crucial for precise staging.
  ○ Two major frameworks exist: EOSS stages obesity based on comorbidities and functional impairment, while DMO identifies early/preclinical obesity; however, their concordance and classification differences have not been systematically compared.
- What does this study add?
  ○ We demonstrate that EOSS emphasizes established obesity‐related disease, whereas DMO captures a broader spectrum of early or subclinical organ dysfunction, revealing distinct classification patterns within the same BMI‐defined population.
  ○ Combining BMI with anthropometric measures can reclassify up to ~50% of individuals, illustrating the sensitivity of early‐stage assignment to diagnostic thresholds.
- How might these results change the direction of research or the focus of clinical practice?
  ○ This study highlights complementary features of EOSS and DMO that may support future strategies for multidimensional obesity assessment.
  ○ We provide evidence for harmonizing obesity classification beyond BMI, emphasizing the need for multidimensional assessment in both research cohorts and routine clinical practice.

## 1 Introduction

Obesity is a chronic, complex, and relapsing multisystem disease recognized by the World Health Organization (WHO) and numerous medical societies [1, 2, 3, 4]. Early diagnosis and appropriate staging are crucial to guide personalized treatment strategies and improve clinical outcomes [5, 6]. Defining disease severity, however, remains challenging. Within the ICD framework, the diagnosis and grading of obesity still rely solely on body mass index (BMI) thresholds [4]. BMI‐based classification may misclassify adiposity and does not adequately capture organ dysfunction, functional impairment, or individual health risk [7].

As proposed by the European Association for the Study of Obesity (EASO) and others [1, 2, 3, 8, 9], it is recommended to assess medical, functional, and psychological impairments alongside anthropometric measures to better identify individual health risks and avoid undertreatment [1, 8, 10, 11, 12, 13, 14]. The Edmonton Obesity Staging System (EOSS), introduced in 2009 [15], exemplifies this approach by proposing a simple clinical and functional staging system. EOSS describes the morbidity and functional limitations associated with excess weight on a five‐point ordinal scale, where Stage 0 indicates no apparent risk factors; Stage 1 reflects subclinical risk factors; Stage 2 involves established chronic diseases; Stage 3 signifies end‐organ damage; and Stage 4 denotes severe disabilities resulting from obesity‐related conditions. Importantly, EOSS has been associated with increased cardiovascular and all‐cause mortality [14].

In 2025, the Lancet Commission on Clinical Obesity proposed a new Diagnostic Model for Obesity (DMO), emphasizing the impact of excess adiposity on organ and tissue function [12]. DMO recommends using BMI (≥ 30 kg/m^2^) only for initial screening. Comprehensive assessment of obesity should confirm excess fat accumulation by either direct measurement of body fat or by at least one anthropometric criterion, including waist circumference (WC), waist to hip ratio (WHR), or waist to height ratio (WHtR). Only in individuals with very high BMI (> 40 kg/m^2^) can excess adiposity be pragmatically assumed, and further confirmation is not required. DMO further differentiates between preclinical and clinical obesity. Preclinical obesity is characterized by excessive fat accumulation without current organ dysfunction or limitations in daily activities but with an increased risk for obesity‐related diseases. In contrast, clinical obesity involves measurable organ or tissue dysfunction and/or significant age‐adjusted restrictions in daily functioning resulting from excess adiposity.

The primary aim of this study was to perform a cross‐classification of individuals with obesity using DMO and EOSS to elucidate how these frameworks capture the heterogeneity of obesity‐related disease and adiposity beyond BMI alone. Building on the DMO framework, which requires confirmation of excess adiposity beyond BMI alone, we hypothesized that combining BMI with additional anthropometric measures would result in reclassification across staging systems. We therefore investigated how various anthropometric parameters affect obesity categorization. To accomplish this, we utilized data from the UK Biobank (UKBB) [16], a large, well‐characterized prospective cohort comprising ~500,000 participants. This extensive dataset enables a comprehensive comparison of obesity classification systems and their underlying conceptual differences.

## 2 Methods

### 2.1 Cohort Description

The UKBB cohort consists of a total of 500,000 participants, as described elsewhere [16]. Only participants self‐identifying as “British,” “Irish,” or “any other White background” were included in the analysis. Participants of other ethnicities (e.g., “Asian or Asian British,” “Black or Black British,” “Mixed,” or “Chinese”) were excluded due to small sample sizes and to further minimize potential ethnic bias. Individuals with diagnoses such as cancer (UKBB field ID 2453), alcoholic liver disease (UKBB field ID 131658), alcoholism (International Statistical Classification of Diseases, Tenth Revision [ICD‐10]: F10), drug abuse (ICD‐10: F19), pregnancy at the time of data collection (UKBB field ID 3140), and toxic liver disease (UKBB field ID 131660) were excluded. This approach resulted in a cohort of *N* = 411,445 participants (54% women, age 56.5 ± 8 years). According to the WHO BMI classification [17], participants were categorized as having underweight (*N* = 1933), normal weight (*N* = 134,884), overweight (*N* = 175,792), and obesity (*N* = 98,836). A detailed description of the cohort is provided in Table S1.

### 2.2 Classification Based on the Modified EOSS

The criteria for assigning EOSS stages were adopted from the Clinical Obesity Chronic Disease Dashboard [18], a modified version from the original EOSS framework [15] providing detailed operational definitions of obesity‐related complications rather than the general concept described in the original framework. For the primary analyses, obesity was defined as BMI ≥ 30 kg/m^2^ according to WHO criteria. Alternative BMI and anthropometric thresholds were evaluated separately in exploratory sensitivity analyses as described in Section 3.4. Clinical parameters, including cutoffs, ICD codes, and UKBB field numbers used for EOSS implementation, are provided in Table S2; concomitant medications are listed in Table S3. International Classification of Diseases, Ninth Revision (ICD‐9) codes provided by the Clinical Obesity Chronic Disease Dashboard were translated into ICD‐10, as documented in Table S4. Hypertension and diabetes were defined according to the Canadian Primary Care Sentinal Surveillance Network (CPCSSN) [19], with HbA1c > 7% used as the laboratory criterion for diabetes due to unavailable fasting glucose data in the UKBB. CPCSSN hypertension was set to missing if any of the data matched any of the CPCSSN exclusion criteria. The estimated glomerular filtration rate (eGFR) was computed using the CKD‐EPI creatinine equation [20]. Although the original EOSS framework considers both mental health and functional limitations for staging, the modified EOSS [18] did not include these. In our analysis, we incorporated activities of daily living (ADL), by combining self‐reported mobility and daily activity limitations into a single ADL category, and mental health, assessed using the General Anxiety Disorder‐7 (GAD‐7) [21] and Patient Health Questionnaire‐9 (PHQ‐9) [22] scores, into the EOSS classification. Each diagnostic criteria generated a score (*N* = 10), and the highest score determined the final EOSS stage. Participants with < 5 available scores were excluded, mainly due to missing kidney, ADL, and mental health scores.

### 2.3 Classification Based on the Lancet Commission's Diagnostic Model for Obesity

For the primary analyses, obesity was defined as BMI ≥ 30 kg/m^2^ and body fat percentage > 25% for males or > 30% for females, following the Lancet Commission [12]. Alternative BMI and anthropometric thresholds were evaluated separately in exploratory sensitivity analyses as described in Section 3.4. Participants were classified as having preclinical or clinical obesity based on evidence of organ or tissue dysfunction, including signs, symptoms, or diagnostic test results. Clinical obesity was assigned when any diagnostic criterion was met. Diagnostic criteria, including parameter thresholds, comorbidities, ICD codes, and UKBB field numbers, are provided in Table S5. ADL were included in the DMO classification using the same self‐reported measures as in EOSS. Each organ system was scored 1 if any diagnostic criterion was met, 0 otherwise; participants with any score of 1 were classified as having clinical obesity.

### 2.4 Statistical Analyses

Data are presented as mean ± SD. Correlations between clinical obesity‐related parameters were assessed using univariate Spearman correlation analyses. Multiple testing corrections were applied by controlling the false discovery rate (FDR) appropriate for the sample size. UKBB data analysis was performed in R (v4.4.0) using the Research Analysis Platform (RAP), which is enabled by DNAnexus technology and powered by Amazon Web Services (AWS).

## 3 Results

### 3.1 Classification of Obesity Grades According to EOSS

**FIGURE 1:**
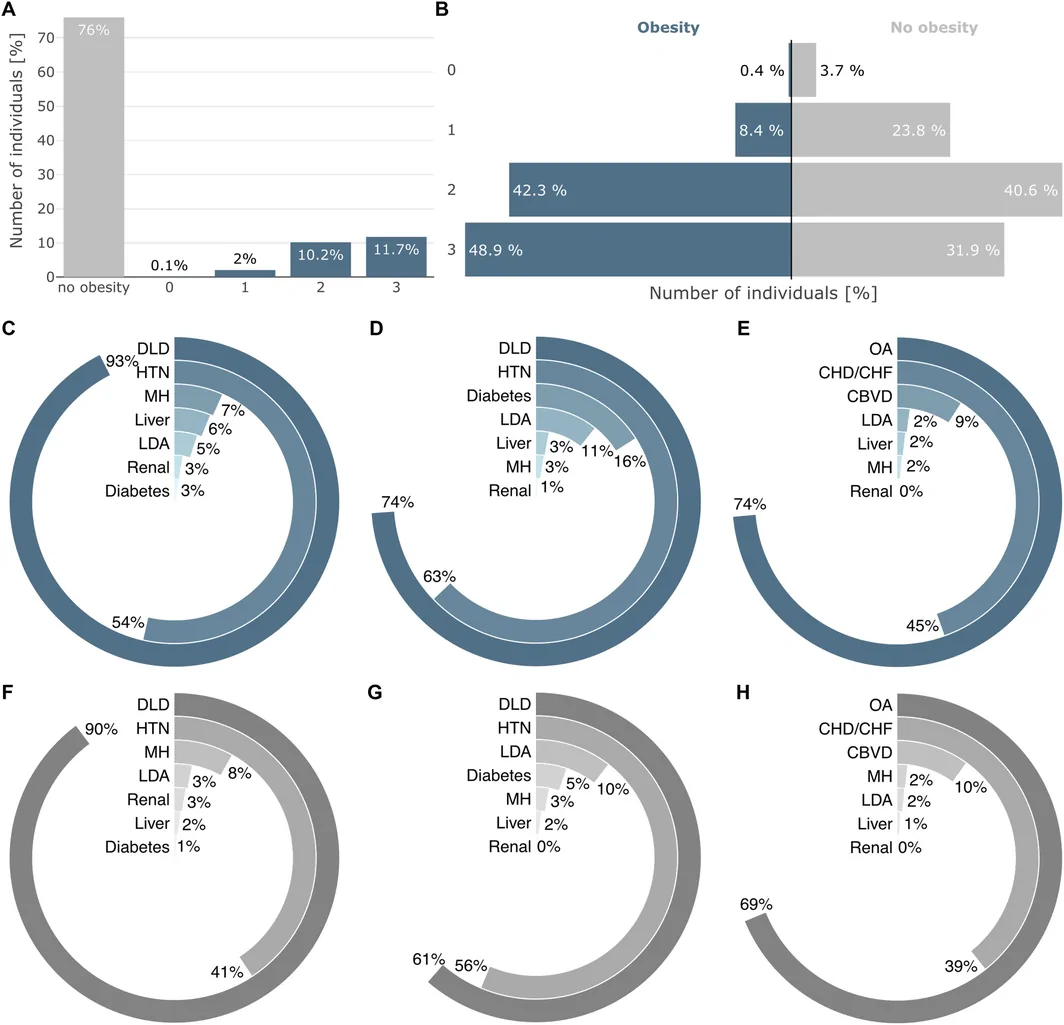
Edmonton Obesity Staging System (EOSS) and diagnostic criteria distribution. (A) Classification of the UKBB cohort according to EOSS. The number of individuals is presented as percentages for those without obesity (BMI < 30 kg/m^2^; gray), as well as for individuals with obesity (blue), differentiated into EOSS Stages 0–3. (B) Percentage distribution of the four EOSS stages among individuals with and without obesity. (C‐E) Percentage distribution of the occurrence of comorbidities contributing to the classification of patients with obesity into EOSS (C) Stage 1, (D) Stage 2, and (E) Stage 3. (F–H) Percentage distribution of diagnostic criteria in individuals without obesity, classified into (F) Stage 1, (G) Stage 2, and (H) Stage 3. In panels C–H, only comorbidities that defined stage allocation are shown; comorbidities with nondetermining scores are not included. Percentages indicate their relative contribution. CBVD: cerebrovascular disease; CHD/CHF: coronary heart disease or congestive heart failure; DLD: dyslipidemia; HTN: hypertension; LDA: limitations of daily activities; MH: mental health; OA: osteoarthritis. [Color figure can be viewed at wileyonlinelibrary.com]

To investigate the distribution of obesity‐related disease severity within the UKBB cohort, participants were categorized according to the modified EOSS. Overall, 24% of UKBB probands were classified as having obesity (BMI ≥ 30 kg/m^2^) (Figure 1A). When further stratifying individuals with obesity (Figure 1B), only 0.4% fell into EOSS Stage 0, indicating no apparent health impairments despite obesity. An additional 8.4% exhibited obesity‐related subclinical risk factors (Stage 1). Most participants with obesity were assigned to more advanced stages: 42.3% to Stage 2 with established obesity‐related chronic diseases and 48.9% to Stage 3 with end‐organ damage. EOSS Stage 4 could not be assigned because the UKBB lacks data on end‐stage disabilities typically observed in clinical settings rather than in population‐based cohorts.

Although originally designed for patients with obesity, we also applied EOSS to participants with BMI < 30 kg/m^2^ in an exploratory manner to compare comorbidity burden across BMI categories (Figure 1B). Compared to individuals with obesity, those without obesity were more often categorized into lower modified EOSS stages (+3.3% in Stage 0, +15.4% in Stage 1) and less frequently into higher stages (−1.7% in Stage 2, −17% in Stage 3).

We further examined the prevalence of comorbidities contributing to stage assignment. Only conditions directly determining stage assignment are displayed, while a complete overview is provided in Table S6. In participants with obesity, dyslipidemia and hypertension were the most common conditions contributing to Stage 1 (93% and 54%) (Figure 1C) and Stage 2 (74% and 63%) (Figure 1D). Limitations in ADL and mental health impairments were observed in 5% and 7% of individuals in Stage 1 and in 11% and 3% in Stage 2. Liver‐related diseases were present in 6% of participants in Stage 1 but only 3% in Stage 2, whereas type 2 diabetes (T2D) increased from 3% to 16% in these stages. In Stage 3 (Figure 1E), defined by severe diseases, osteoarthritis predominated (74%), followed by coronary artery disease or congestive heart failure (45%) and cerebrovascular disease (9%). Renal impairment remains relatively uncommon across all modified EOSS stages. Among participants without obesity, the relative order of comorbidity prevalence across stages remained largely similar, although absolute frequencies were lower (Figure 1F–H).

### 3.2 Severity of Obesity According to the Lancet Commission's Diagnostic Model for Obesity

**FIGURE 2:**
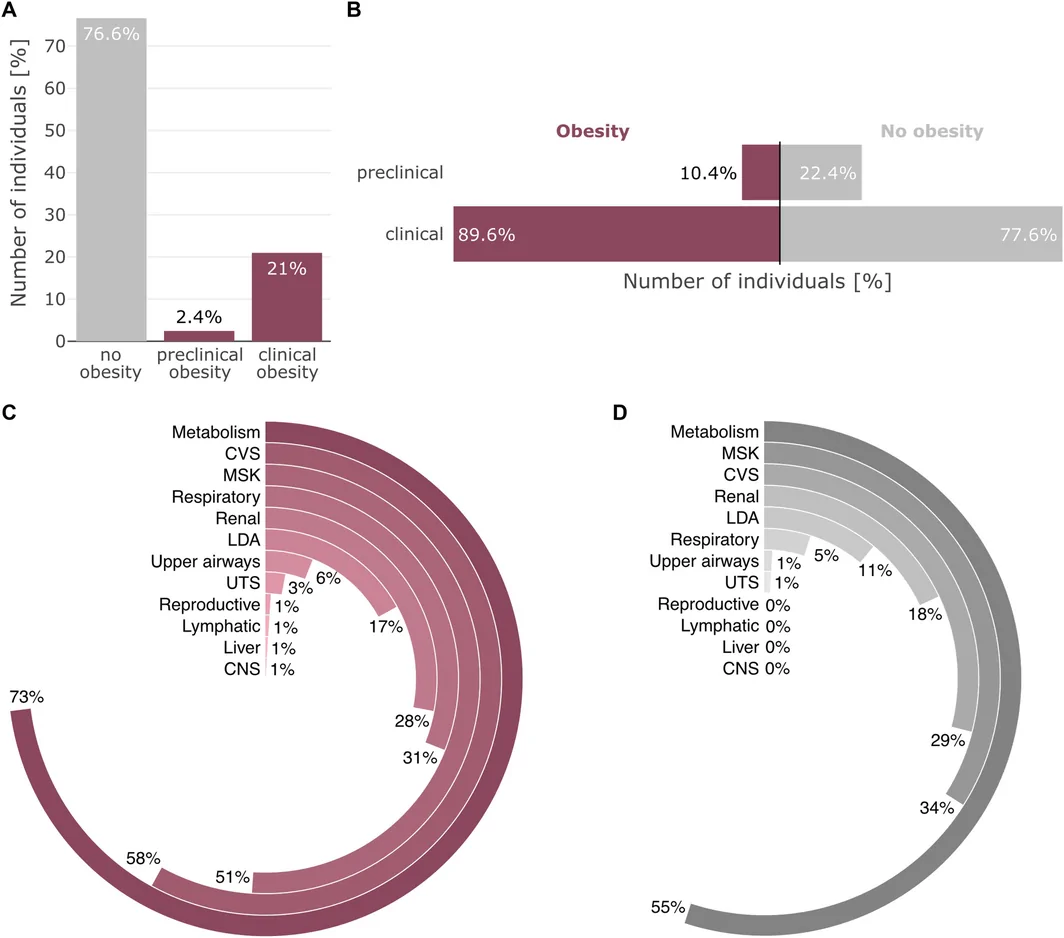
Diagnostic Model for Obesity (DMO) classification and diagnostic criteria distribution. (A) Classification of the UKBB cohort according to DMO. The number of individuals is presented as percentages for those without obesity (BMI < 30 kg/m^2^ and body fat ≤ 25% in males; ≤ 30% in females; gray), as well as for individuals with obesity (red), differentiated into preclinical and clinical obesity. (B) Percentage distribution of preclinical and clinical classifications among individuals with and without obesity. (C) Percentage distribution of diagnostic criteria contributing to the classification of patients with obesity as having clinical obesity. (D) Percentage distribution of diagnostic criteria in individuals without obesity. In panels C and D, percentages indicate their relative contribution. CNS: central nervous system; CVS: cardiovascular system; LDA: limitations of daily activities; MSK: musculoskeletal; UTS: urinary tract system. [Color figure can be viewed at wileyonlinelibrary.com]

Applying the Lancet Commission's DMO classification, 23.4% of participants were identified as having obesity (BMI ≥ 30 kg/m^2^, body fat > 25%/30% [M/F]) (Figure 2A). Among individuals with obesity, clinical obesity occurred about 8.6 times more frequently than preclinical obesity (Figure 2B). Individuals with obesity showed a 12% higher rate of clinical obesity classification than those without obesity, reflecting differences in the prevalence of organ or tissue dysfunction and ADL impairment (Figure 2B). The most frequent criteria contributing to clinical obesity classification (Figure 2C) were metabolic (73%), cardiovascular (58%), and musculoskeletal dysfunction (51%). Other contributors included respiratory (31%) or renal dysfunction (28%) and LDA (17%). In individuals without obesity (Figure 2D), these criteria occurred at substantially lower frequencies, particularly for cardiovascular (−29%), respiratory (−26%), metabolic (−18%), musculoskeletal (−17%), and renal impairments (−10%) and LDA (−6%). Dysfunction of the liver, central nervous system, and urinary, lymphatic, or reproductive systems was rare or absent in both groups. A detailed overview of diagnostic criteria and phenotypic characteristics for each classification is provided in Table S7.

### 3.3 Differences Between the Classification Systems in Defining Obesity Severity

**FIGURE 3:**
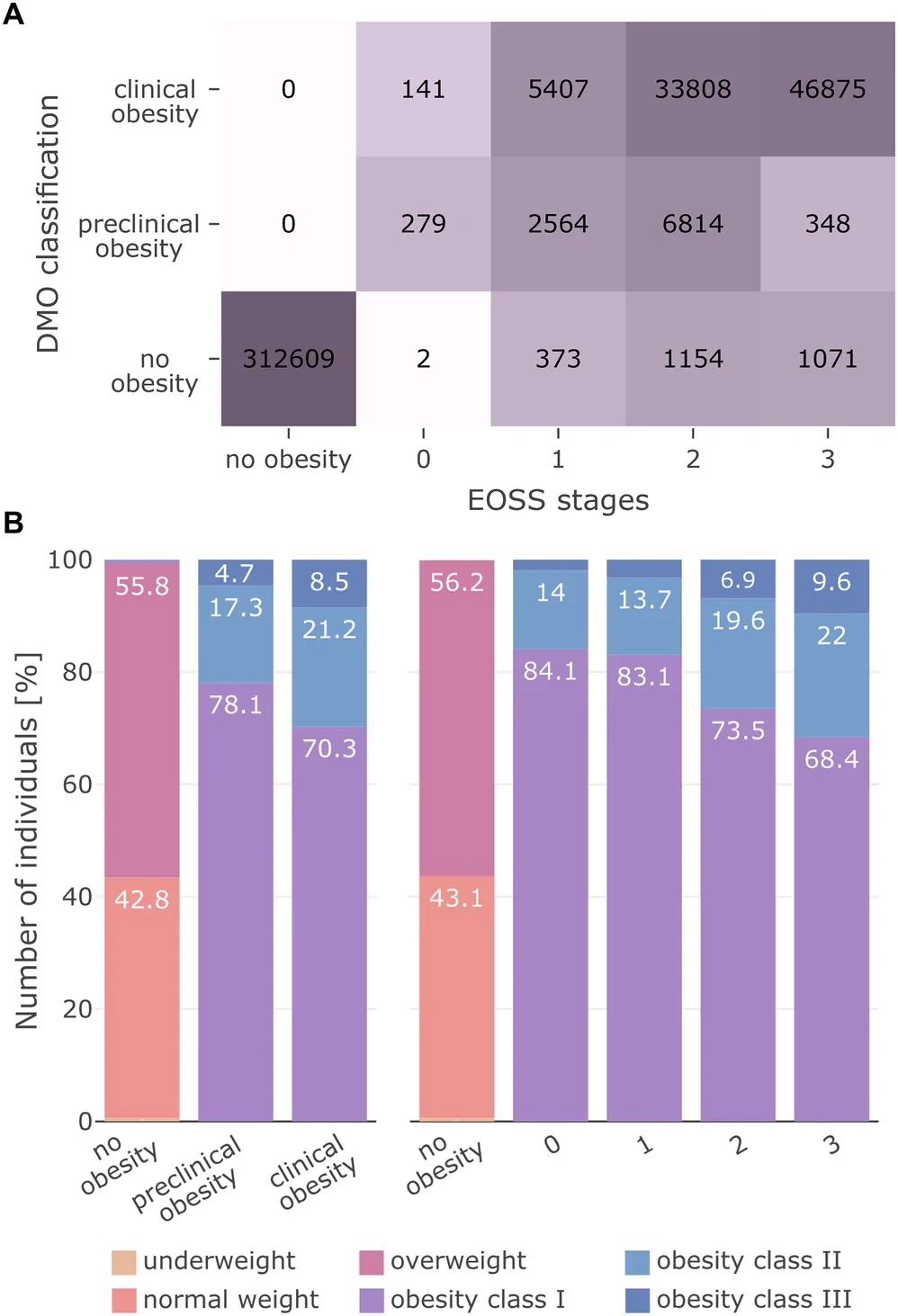
Patient and BMI class distribution across the diagnostic classification systems. (A) Heat map displaying the absolute number of patients in the UKBB, with a comparative overview of patient counts across EOSS and DMO classification groups. (B) Percentage distribution of World Health Organization‐defined BMI classes within each EOSS and DMO classification group. [Color figure can be viewed at wileyonlinelibrary.com]

To compare how participants are classified by the modified EOSS and DMO, Figure 3A presents the absolute numbers of participants assigned to each category. Due to the different criteria for defining obesity (EOSS: BMI ≥ 30 kg/m^2^; DMO: BMI ≥ 30 kg/m^2^ and body fat > 25%/30% [M/F]), some individuals classified as having no obesity by DMO were categorized into EOSS Stages 0–3. Notably, a substantial proportion of these individuals were assigned to Stage 2 (*N* = 1154) and Stage 3 (*N* = 1071). Although many participants were assigned to comparable classification categories (e.g., EOSS Stages 1–3 and clinical obesity), differences remain. Of the individuals classified as having preclinical obesity by DMO (*N* = 10,005), only 279 were assigned to EOSS Stage 1. In contrast, most participants in EOSS Stage 0 (279 of 420) were classified as having preclinical obesity by DMO, indicating that EOSS classifies fewer individuals to the lowest‐stage category.

To evaluate how WHO BMI categories reflect obesity‐related health status, we examined their distribution across DMO and EOSS classifications (Figure 3B). Higher obesity classes were associated with a greater prevalence of advanced modified EOSS stages and clinical obesity in DMO. However, a considerable proportion of individuals with obesity Class II or III remained categorized as having preclinical obesity (DMO) or remained in the lower EOSS stages (0–1).

Additionally, we examined obesity‐related clinical parameters not utilized for classification across obesity severity levels (Figures S1 and S2). Obesity severity defined by DMO and EOSS was more closely associated with age and MRI‐assessed visceral adipose tissue (VAT) volume than with BMI or other anthropometric measures. Age increased with higher obesity stages but showed little association with fat distribution parameters, suggesting that higher disease burden was associated with increasing age despite limited differences in fat distribution parameters. Although MRI‐assessed VAT levels strongly correlate with BMI and other fat distribution measures, VAT volume showed a clearer stepwise increase across obesity severity stages. In contrast, body weight, WC, WHR or WHtR, and MRI‐assessed subcutaneous adipose tissue (SAT) volumes remained relatively stable.

### 3.4 Effect of Adding Anthropometric Criteria to Categorize Obesity Severity

The choice of clinical parameters and cutoff values for defining obesity remains debated. Recent recommendations, including those from the Lancet Commission on Clinical Obesity and EASO, suggested incorporating measures of excess adiposity beyond BMI [1, 12]. Specifically, the Lancet Commission suggests incorporating either assessments of body fat or at least one anthropometric criterion such as WC, WHR, or WHtR, in addition to BMI. For individuals with BMI ≥ 40 kg/m^2^, they proposed that excess adiposity may be assumed pragmatically.

As exploratory sensitivity analyses, we evaluated several proposed parameters and cutoff thresholds for their effect on classification outcomes. Parameters tested included (I) BMI ≥ 30 kg/m^2^ alone (in line with WHO [23]) or the Lancet Commission recommendation [12] BMI ≥ 40 kg/m^2^ or BMI ≥ 30 kg/m^2^ in combination with (II) body fat > 25%/30% (M/F), (III) WC > 102/88 cm (M/F), (IV) WHR > 0.9/0.85 (M/F), or (V) WHtR > 0.5. Furthermore, as recommended by EOSS and others [1, 11, 14] a lower (VI) BMI threshold of ≥ 25 kg/m^2^ was also tested, either alone or in combination with the same additional parameters suggested by the Lancet Commission: (VII) body fat, (VIII) WC, (IX) WHR, or (X) WHtR. The European guidelines for obesity management [10] additionally proposed using (XI) WC thresholds of ≥ 94/80 cm (M/F) alone or/and (XII/XIII) combined with BMI ≥ 25 kg/m^2^. Finally, (XIV) BMI ≥ 30 kg/m^2^ or BMI ≥ 25 kg/m^2^ in combination with WHtR > 0.5 was tested following the guidance of EASO [1].

**FIGURE 4:**
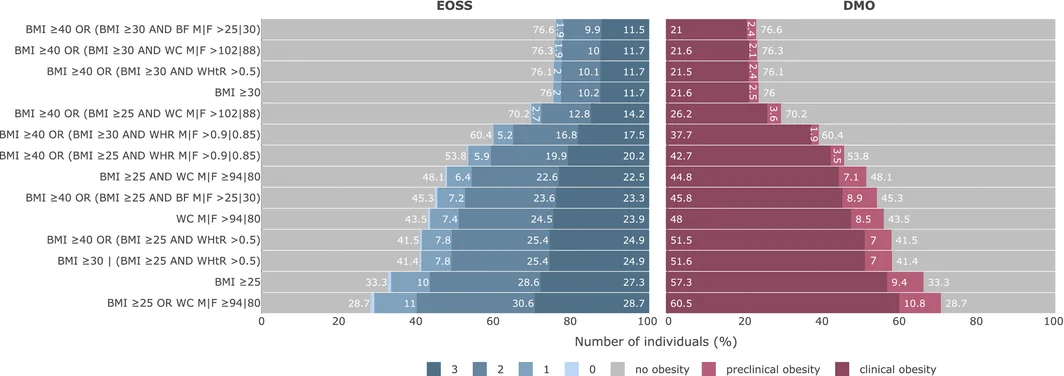
Impact of clinical parameter selection on diagnostic classification systems. Assessment of the impact of different clinical parameter cutoffs on the classification of UKBB participants into no obesity and obesity groups for EOSS (left) and DMO (right). Different cutoffs and their combinations were evaluated for BMI (kg/m^2^), waist circumference (WC, cm), waist to height ratio (WHtR), and waist to hip ratio (cm), with certain cutoffs being gender‐specific, adjusted separately for males (M) and females (F). Values are shown only if > 1%. [Color figure can be viewed at wileyonlinelibrary.com]

As shown in Figure 4, the proportion of participants classified with obesity varied substantially depending on the chosen parameter combinations and thresholds, with consistent patterns across both diagnostic systems. More restrictive criteria, particularly BMI ≥ 30 kg/m^2^ combined with (II) body fat, (III) WC, or (V) WHtR, produced largely stable classifications comparable with (I) BMI ≥ 30 kg/m^2^ alone. In contrast, combining BMI with (IV) WHR resulted in the largest increase in individuals classified with obesity.

The highest prevalence of obesity was observed using (VIII) BMI ≥ 25 kg/m^2^ or WC ≥ 94/80 cm (M/F), followed by (VI) BMI ≥ 25 kg/m^2^ alone. Under the BMI ≥ 25 kg/m^2^ threshold, additional parameters influenced classifications more strongly, with WC and WHR yielding comparatively lenient results, while body fat and WHtR reduced obesity prevalence more substantially.

## 4 Discussion

Obesity is a complex and chronic condition that remains substantially underdiagnosed despite its well‐documented health risks. In the UKBB, only 9.8% of participants had a recorded ICD‐10 diagnosis of obesity, underscoring the gap between clinical documentation and the actual burden of adiposity. In this study, we therefore performed a cross‐classification using the modified EOSS and DMO frameworks to evaluate how these systems classify adiposity‐related disease burden.

Approximately one‐quarter of participants were classified as having obesity under both frameworks. However, most individuals with obesity were assigned to advanced stages of disease classification, with nearly half of participants reaching EOSS Stage 3 and clinical obesity predominating within the DMO classification. Similar distributions have been reported in other cohorts, although differences between the UKBB, the Northern Alberta Primary Care Research Network [18], and the All of Us cohort [24] likely reflect variation in age structure, recruitment strategies, and underlying health status. Nevertheless, the high proportion of individuals classified in advanced disease stages reflects the substantial burden of obesity‐related conditions in this population.

When applying both classification systems to individuals with and without obesity, participants with adiposity showed a higher prevalence of advanced disease stages, whereas individuals without obesity were predominantly assigned to lower stages. These findings highlight the coexistence of excess adiposity and multisystem disease burden across BMI categories. However, the application of EOSS to individuals with BMI < 30 kg/m^2^ should be interpreted with caution, as the system was not designed to define disease presence. In this context, analyses across BMI groups reflect overall comorbidity burden rather than disease strictly causally attributable to obesity.

Both frameworks identify metabolic and cardiovascular impairments as the most prevalent domains among individuals with obesity, consistent with previous reports [18, 25]. They differ, however, in operationalization. The modified EOSS uses defined clinical thresholds and established end‐organ damage, including diabetes, hypertension, dyslipidemia, and cardiovascular disease. In contrast, DMO captures broader metabolic dysfunction, such as hyperglycemia and abnormal lipid profiles, without using diabetes as a direct staging determinant. DMO also includes musculoskeletal, respiratory, upper airway, central nervous system, urinary, and lymphatic dysfunction, representing a broader morbidity spectrum. In EOSS, musculoskeletal disease mainly reflects osteoarthritis in Stage 3, while mental health parameters are uniquely incorporated in EOSS.

Renal impairment further illustrates differences in the detection of early abnormalities between the two systems. In the DMO framework, renal abnormalities were present in more than a quarter of individuals with clinical obesity, whereas ≤ 3% of participants met renal criteria in the modified EOSS. This discrepancy likely reflects the broader inclusion of mild or early renal abnormalities in DMO, whereas the modified EOSS relies on stricter thresholds based on eGFR and albuminuria, thereby capturing more advanced disease. Liver disease was identified at low frequencies in both frameworks, which may reflect limited availability of diagnostic assessments in population‐based cohorts. The modified EOSS identifies hepatic involvement mainly through abnormal liver enzymes or diagnosed liver disease, while DMO includes metabolic dysfunction‐associated steatotic liver disease and hepatic fibrosis as diagnostic entities. Because these conditions frequently require imaging or biopsy, they are rarely detected clinically [26].

Taken together, the modified EOSS emphasizes clinically established disease and organ damage, whereas DMO adopts a broader perspective capturing functional limitations and organ dysfunction across multiple systems. Consequently, DMO identifies a wider spectrum of obesity‐related impairments, while EOSS emphasizes clinically established disease and higher‐stage disease manifestations. In our cohort, fewer participants were classified as Stage 0 under EOSS than as having preclinical obesity under DMO, likely reflecting the stricter metabolic and psychological criteria required to meet the EOSS Stage 0 definition, whereas DMO allows classification as preclinical obesity despite the presence of early or subclinical abnormalities. This illustrates that the prevalence of “metabolically healthy obesity” depends on the criteria used to define early abnormalities. Cross‐classification further revealed that a subset of individuals classified as nonobese in DMO were assigned to advanced EOSS stages, illustrating the conceptual differences between both frameworks. Recognizing these complementary characteristics, a recent proposal suggested combining the DMO diagnostic definition with EOSS staging to integrate multidimensional diagnosis with severity stratification [27]. Such an approach may help distinguish individuals requiring active obesity treatment from those with lower disease burden while preserving the prognostic value of obesity severity staging. An important next step will be to evaluate the predictive performance of obesity classification systems with respect to clinically meaningful outcomes. Approaches similar to Atlantis et al. [28], including modeling of health service utilization, incident disease, and pharmacotherapy use, as well as assessment of discrimination and reclassification, may provide further insight into associations with clinically meaningful outcomes.

Both frameworks share limitations. Liver disease and several organ system impairments appear underrepresented, which may reflect limited detection of these conditions in population‐based cohorts. In addition, neither system incorporates lifestyle‐related risk factors, such as dietary patterns, smoking, or alcohol consumption, despite their strong influence on obesity‐related comorbidities [29]. Cancer is also not included in either framework, although evidence from the UKBB demonstrates strong associations between both preclinical and clinical obesity in the DMO framework and multiple obesity‐related cancers [30].

A foundational step for any classification system is to distinguish between individuals with and without obesity, as BMI alone does not capture fat distribution or composition. Consistent across both EOSS and DMO classifications, our analysis revealed substantial heterogeneity within WHO BMI categories: although higher obesity classes generally corresponded to more advanced disease stages, a considerable proportion of individuals with Class II or III obesity were still categorized as having preclinical obesity or remained in lower EOSS stages (0–1). These findings highlight that individuals with similar BMI can exhibit different metabolic and functional profiles.

Current guidelines therefore recommend complementing BMI with measures of abdominal fat accumulation such as WC, WHtR, or WHR [1, 8, 10, 11, 31]. Our findings underline that diagnostic thresholds are important, as up to ~50% of individuals are being reclassified depending on the applied definition. However, combining BMI ≥ 30 kg/m^2^ with WC, body fat, or WHtR resulted in only minor deviations from BMI ≥ 30 kg/m^2^ alone, suggesting limited additional value of these parameters once a clear obesity threshold is reached. In contrast, combining BMI ≥ 30 kg/m^2^ with WHR produced the least restrictive classifications. This likely reflects that WHR depends strongly on both waist and hip circumference and may therefore capture body shape rather than visceral fat accumulation. At lower BMI thresholds (≥ 25 kg/m^2^), additional anthropometric parameters had a stronger influence on classification, which aligns with the notion that borderline categories are more sensitive to measurement choice [32, 33]. These findings suggest that WC or WHR may classify a broader range of individuals at lower BMI thresholds, whereas body fat or WHtR result in different classification patterns with greater restriction. These findings support current calls for harmonization of cutoff definitions [12, 33], emphasizing the need for evidence‐based standardization to avoid inconsistencies in obesity classification across clinical and research settings.

Finally, obesity severity in our cohort was more strongly associated with age and MRI‐derived VAT than with BMI or other standard anthropometric measures. Age increased across obesity severity categories, whereas VAT showed a clear stepwise increase across stages, suggesting that imaging‐derived measures may provide additional information beyond conventional anthropometric criteria.

A limitation of this study is that the UKBB does not allow assessment of whether comorbidities are directly attributable to adiposity. We therefore assumed that all documented conditions were obesity‐related, which may overestimate associations between adiposity and comorbidities. This aligns with the EOSS approach where establishing causality between obesity and comorbidities is not required for stage assignment. In contrast, DMO incorporates obesity‐related causality as part of its diagnostic framework, representing a different conceptual approach.

Another limitation concerns the operationalization of the DMO criteria, which required adaptation for implementation in a population‐based cohort. Similarly, we applied a modified EOSS framework based on the Clinical Obesity Chronic Disease Dashboard [18] rather than the original EOSS [15], as operational definitions were required for cohort‐based analyses; similar adaptations have been used previously [14, 18, 34]. The application of both frameworks was further constrained by available clinical and laboratory data. Importantly, this study focused on cross‐classification and concordance between obesity frameworks; therefore, associations with longitudinal clinical outcomes, predictive performance, and potential clinical utility were not assessed. Functional limitations were assessed using self‐reported measures of daily activity and mobility, which may introduce reporting bias. Finally, the UKBB cohort is not fully representative of the general population and tends to include healthier volunteers and is composed predominantly of individuals of White European ancestry, which may limit generalizability. As BMI, adiposity, and obesity‐related complications differ across ethnic groups, future studies should evaluate ethnicity‐specific cut‐offs within both frameworks.

## 5 Conclusion

Our comparison of the modified EOSS and the DMO frameworks highlights their complementary roles in characterizing obesity‐related disease burden. While EOSS provides structured staging based on clinically established obesity‐related conditions, DMO captures a broader spectrum of functional limitations and early organ dysfunction across multiple systems. The observed discordance between both frameworks and the substantial heterogeneity within BMI categories further emphasize that BMI alone does not adequately reflect obesity severity. In addition, our findings underline the influence of diagnostic thresholds and fat distribution measures on obesity classification. Integrating the complementary perspectives of EOSS and DMO, together with harmonized diagnostic criteria, may support future development and evaluation of multidimensional obesity classification approaches.

## Author Contributions

Conceptualization: M.B. and A.H.; Methodology: M.B., A.H., and T.H.; Validation: M.B., A.H., and T.H.; Formal analysis: T.H.; Resources: M.B.; Data curation: T.H.; Writing – original draft: A.H., T.H., A.M.S., and M.B.; Writing – review and editing: A.H., A.M.S., T.H., and M.B.; Visualization: A.H., and T.H.; Supervision: A.H. and M.B.; Project administration: A.H.; Funding acquisition: M.B.

## Funding

This work was supported by grants of the Deutsche Forschungsgemeinschaft (DFG), project number 209933838 (SFB1052 “Obesity Mechanisms”: B1 to M.B.) and LeiCeM—Leipzig Center of Metabolism at Leipzig University. The funders had no role in the design of the study, the collection, analyses, or interpretation of data, the decision to publish the results, and the writing of the manuscript.

## Conflicts of Interest

M.B. received honoraria as a consultant and speaker from Abbott, Amgen, AstraZeneca, Bayer, Boehringer Ingelheim, Daiichi–Sankyo, Lilly, MSD, Novo Nordisk, Roche and Sanofi; and reports chairing a Clinical Trial Data Safety Monitoring Board for Boehringer Ingelheim. The other authors declared no conflicts of interest.

## Supporting information


**Figure S1:** Correlation of clinical parameters of the UK Biobank. Spearman correlation matrix of selected clinical parameters. The color scale ranges from red (strong positive correlation, +1) to blue (strong negative correlation, −1). ALAT: alanine aminotransferase; ASAT: aspartate aminotransferase; BMI: body mass index; BP: blood pressure; eGFR: estimated glomerular filtration rate; GGT: gamma‐glutamyl transferase; HbA1c: hemoglobin A1c; HDL: high‐density lipoprotein; LDL: low‐density lipoprotein; MRI: magnetic resonance imaging; SAT: subcutaneous adipose tissue; uACR: urinary albumin to creatinine ratio; VAT: visceral adipose tissue; WHR: waist to hip ratio; WHtR: waist to height ratio.

**Figure S2:** Comparison of clinical parameters between the two obesity classification frameworks. Box plots of selected clinical parameters displayed for the Edmonton Obesity Staging System (blue colors) and the Diagnostic Model for Obesity (red colors). The plots illustrate the distribution and differences in these variables both between the two assessment methods and within each individual grouping of the systems. BMI: body mass index; MRI: magnetic resonance imaging; SAT: subcutaneous adipose tissue; VAT: visceral adipose tissue; WHtR: waist to height ratio; WHR: waist to hip ratio.

**Table S1:** Detailed cohort description. Phenotypic description of the UK Biobank (UKBB) cohort analyzed, subdivided into the individual World Health Organization (WHO) BMI categories, as well as total population.
**Table S2:** Revised Edmonton Obesity Staging System scoring definitions. Definitions applied for Edmonton Obesity Staging System (EOSS) staging, including clinical parameters, ICD codes, and UK Biobank field numbers for the respective comorbidities, adapted from Swaleh et al. [18]. “n/a” denotes stages not defined for a given comorbidity.
**Table S3:** EOSS scoring concomitant medications. Medications considered for revised EOSS staging, including statins, diabetes, and antihypertensive drugs.
**Table S4:** International Classification of Diseases codes applied for the revised EOSS scoring. ICD‐9 codes as proposed by Swaleh et al. [18] and their corresponding ICD‐10 codes as recorded in the UK Biobank for the respective diseases. All ICD‐10 codes are extracted from the UK Biobank field ID 41270.
**Table S5:** Model for Obesity classification definitions. Definitions applied for the Diagnostic Model for Obesity (DMO) classification, including clinical parameters, ICD codes, and UK Biobank field numbers for the respective diagnostic criteria. All ICD‐10 codes are extracted from the UK Biobank field ID 41270.
**Table S6:** Participant characteristics across EOSS stages. Phenotypic characteristics and comorbidities of individuals stratified by revised EOSS stage.
**Table S7:** Participant characteristics across DMO classes. Phenotypic characteristics and diagnostic criteria of individuals stratified by DMO categories.

## Data Availability

The data used in this study are available from the UK Biobank (https://www.ukbiobank.ac.uk/) upon application. The analyses were performed using the UK Biobank dataset as available in 2025. Due to UKBB policies, the data cannot be publicly shared by the authors.
