# Supplementary material for: Divergent Obesity Classification Between the Edmonton Obesity Staging System and the Lancet Commission Model
Source: Obesity (Silver Spring). 2026 Sep 1;34(10):1989–98. doi: 10.1002/oby.70295 (PMC13615417; doi:10.1002/oby.70295)
Supplement: Supplementary file 1 [file OBY-34-1989-s001.pdf]

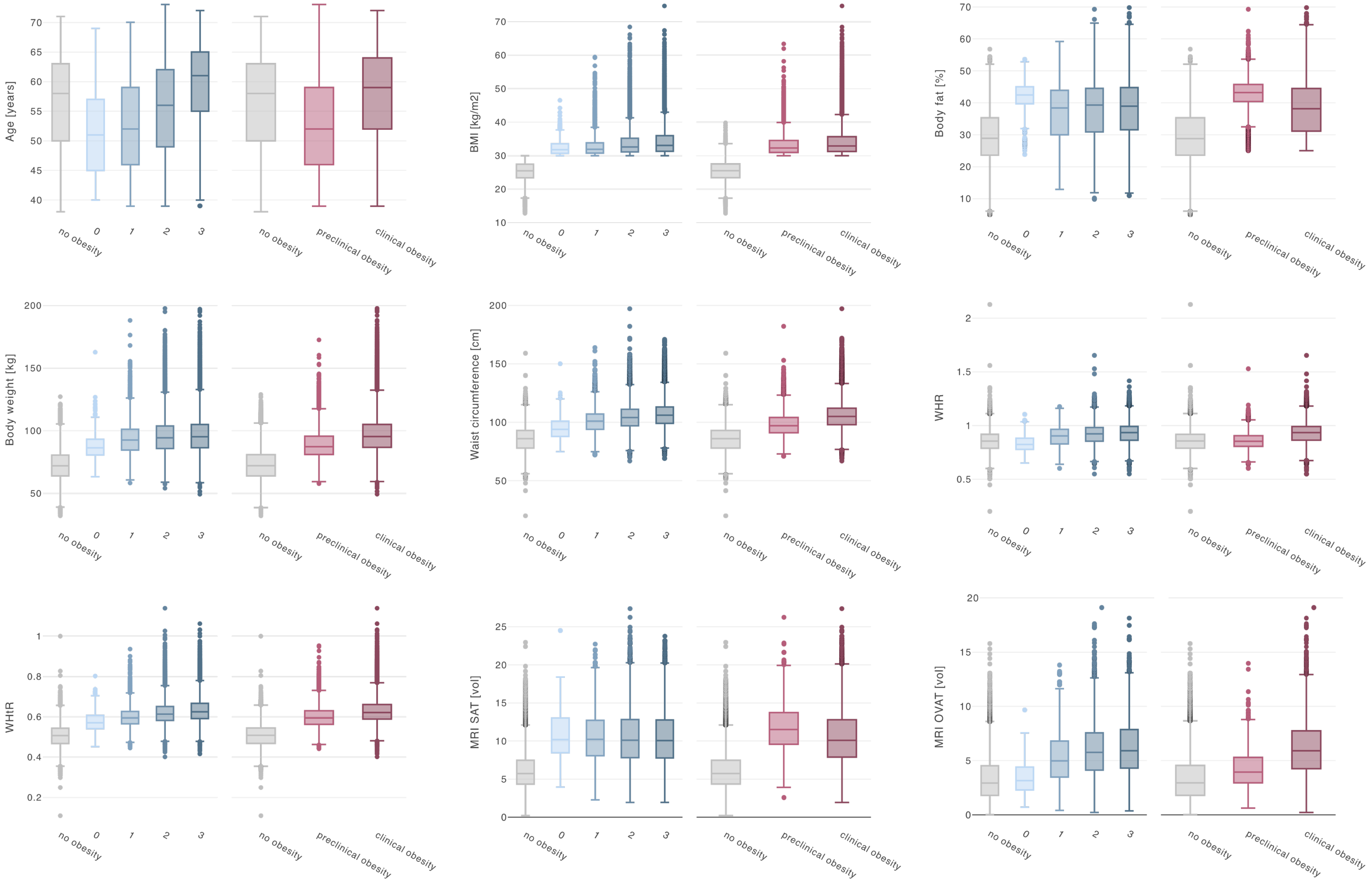

Age [years]
BMI [kg/m2]
Body fat [%]
Body weight [kg]
Waist circumference [cm]
WHR
WHtR
MRI SAT [vol]
MRI OVAT [vol]
no obesity
0
1
2
3
no obesity
preclinical obesity
clinical obesity
