# Supplementary material for: Divergent Obesity Classification Between the Edmonton Obesity Staging System and the Lancet Commission Model
Source: Obesity (Silver Spring). 2026 Sep 1;34(10):1989–98. doi: 10.1002/oby.70295 (PMC13615417; doi:10.1002/oby.70295)
Supplement: Supplementary file 2 [file OBY-34-1989-s002.pdf]

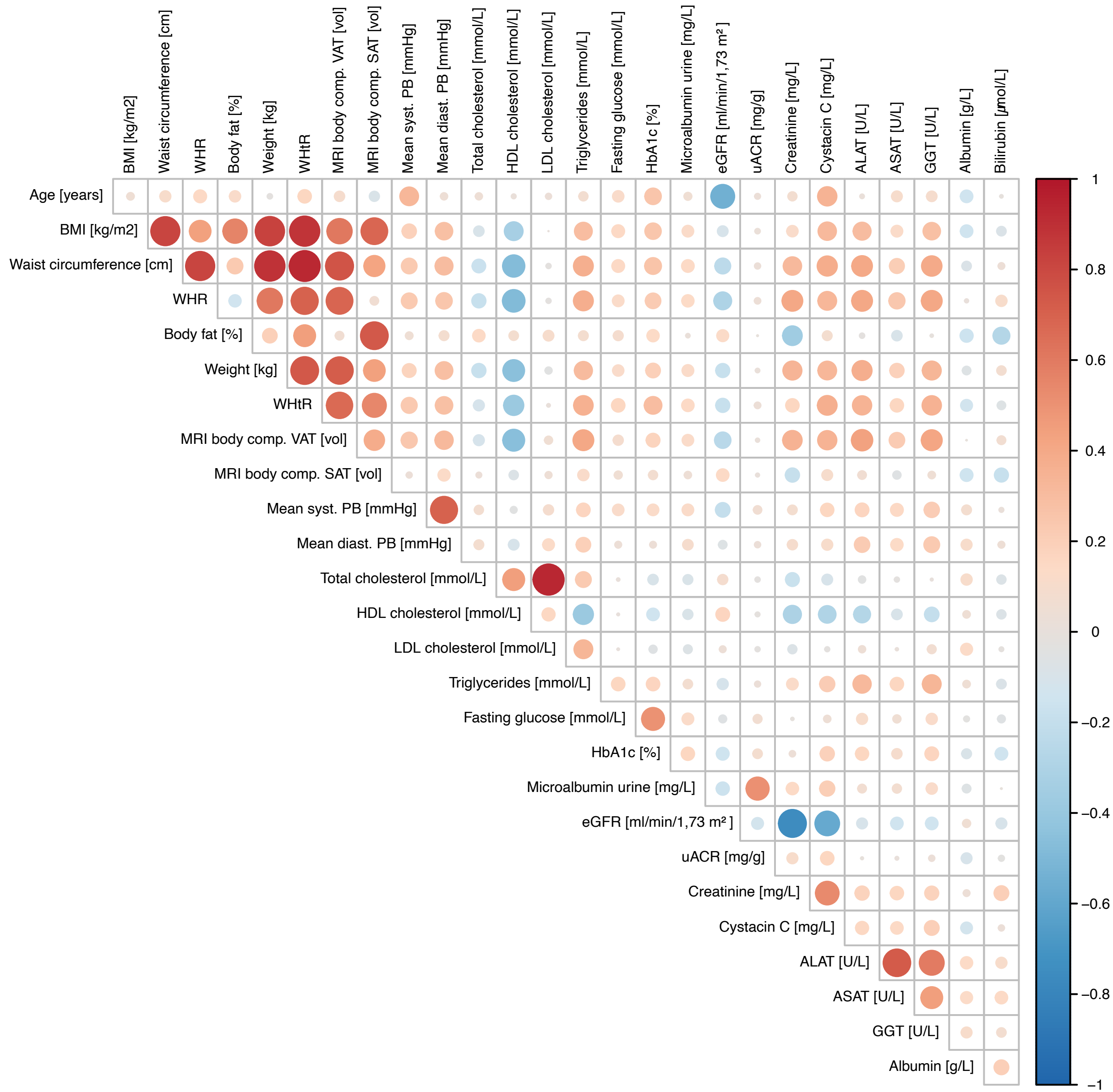

Age [years]
BMI [kg/m2]
Waist circumference [cm]
WHR
Body fat [%]
Weight [kg]
WHtR
MRI body comp. VAT [vol]
MRI body comp. SAT [vol]
Mean syst. PB [mmHg]
Mean diast. PB [mmHg]
Total cholesterol [mmol/L]
HDL cholesterol [mmol/L]
LDL cholesterol [mmol/L]
Triglycerides [mmol/L]
Fasting glucose [mmol/L]
HbA1c [%]
Microalbumin urine [mg/L]
eGFR [ml/min/1,73 m²]
uACR [mg/g]
Creatinine [mg/L]
Cystacin C [mg/L]
ALAT [U/L]
ASAT [U/L]
GGT [U/L]
Albumin [g/L]
Bilirubin [µmol/L]
1
0.8
0.6
0.4
0.2
0
−0.2
−0.4
−0.6
−0.8
−1
